# Effects of Napping on Cognitive and Physical Performance in Sleep-Deprived Elite Kung-Fu Athletes

**DOI:** 10.3390/life16020253

**Published:** 2026-02-02

**Authors:** Anis Saddoud, Mohamed Frikha, Mehdi Chlif, Abdulmalek K. Bursais, Anwar Al-Nuaim

**Affiliations:** 1 High Institute of Sport and Physical Education of Sfax, University of Sfax, Sfax 3000, Tunisia; anis.saddoud@isseps.usf.tn; 2 Department of Physical Education, College of Education, King Faisal University, Al-Ahsa 31982, Saudi Arabia; abursais@kfu.edu.sa (A.K.B.); aalnuaim@kfu.edu.sa (A.A.-N.); 3 Research Laboratory: Sports Performance Optimisation, National Center of Medicine and Science in Sports (NCMSS), Tunis 263, Tunisia; mehdi.chlif@isseps.usf.tn; 4 EA 3300, “APS and Motor Patterns: Adaptations- Rehabilitation”, Picardie Jules Verne University, F-80025 Amiens, France

**Keywords:** total sleep deprivation, napping, sports performance, decision-making, kung fu

## Abstract

Background: Total sleep deprivation (TSD) negatively affects athletic performance by impairing mood, anaerobic performance, and decision-making in martial arts athletes. This study examined whether a 45 min nap can alleviate deficits in mood, anaerobic performance, and decision-making caused by TSD in elite Kung-Fu athletes. Methods: Twenty-four elite male Kung-Fu athletes (age: 20.67 ± 1.76 years) participated in four randomised conditions: normal sleep, without sleep deprivation + nap, total sleep deprivation (36 h awake), and TSD + nap. Mood states were assessed in terms of the POMS-f, perceptual responses, decision-making via video-based tasks, barrage test, anaerobic performance through vertical and horizontal jumps, and isometric strength. Results: Sleep deprivation significantly affected mood, with vigour dropping by 53.8% (*p* < 0.001), impaired physical performance, with vertical jump declining from 36.80 ± 6.47 cm to 33.23 ± 6.03 cm (*p* < 0.001), and reduced cognitive function, with decision accuracy declining from 24.00 ± 2.16% to 18.44 ± 2.24% (*p* < 0.001) and reaction time increasing from 0.58 ± 0.08 s to 0.93 ± 0.13 s (*p* < 0.001). Strategic napping significantly enhanced cognitive recovery, with decision accuracy increasing by 14.1% (*p* < 0.001) and reaction time improving by 16.1% (*p* < 0.001). Physical performance showed modest gains, with vertical jump height increasing by 2.4% (*p* < 0.05). Conclusions: Strategic 45 min naps offer superior cognitive recovery compared to physical recovery in sleep-deprived elite Kung-Fu athletes, suggesting that coaches should incorporate strategic napping to enhance decision-making abilities during competitions involving sleep deprivation.

## 1. Introduction

Sleep deprivation poses a significant challenge to athletic performance, particularly in elite athletes who face demanding training schedules and competition-related stressors. Meta-analytic evidence has shown that sleep loss negatively affects various performance areas, including endurance, strength, cognitive processing, and mood regulation [1,2,3]. Acute sleep deprivation reduced aerobic performance, CMJ height, and handgrip strength by 11.4%, 10.9%, and 6%, respectively. Partial sleep deprivation for 4 h can decrease aerobic capacity by 4.1% and jump performance by 5.2% [4,5]. These impairments are particularly pronounced in elite athletic populations, with reviews indicating that insomnia symptoms affect up to 65% of athletes during competitive periods, highlighting the need for effective countermeasures [5,6]. Beyond sports, these findings have critical implications for high-stakes professions, such as military personnel and emergency responders, where sleep disruption and rapid decision-making under pressure present similar challenges [7]. The cumulative effects of chronic sleep deprivation can worsen these issues, leading to a decline in immune function and an increased risk of injury, thereby compromising an athlete’s ability to train and compete optimally [8,9,10].

Cognitive function, which is crucial for athletic success, is particularly vulnerable to sleep deprivation. Evidence shows that a lack of sleep impairs complex cognitive processes that are essential for performance. Decision-making accuracy can decline by 15–50% after 24–36 h of wakefulness, while reaction times slow, vigilance wanes, and executive functions, such as problem solving and tactical planning, deteriorate [11,12,13]. These impairments are especially concerning in combat sports athletes, such as elite Kung-Fu practitioners, whose performance depends on the integration of rapid tactical decision-making with explosive physical demands under intense stress conditions. Theoretically, cognitive–motor integration relies on the prefrontal cortex, which is vital for executive control, attention, and decision-making. Sleep deprivation disrupts prefrontal cortex activity, leading to a reduced capacity for strategic planning and response inhibition under time constraints, which are crucial in dynamic combat scenarios. The unique demands of martial arts, where physical prowess must be combined with strategic thinking, highlight the need for research on sleep-related interventions that can preserve both mental and physical performances. Additionally, emotional regulation in combat sports, such as managing anxiety and aggression during competition, may be compromised by sleep loss, thereby affecting an athlete’s psychological resilience and performance [14,15,16].

Strategic daytime napping has emerged as a promising intervention for mitigating the effects of sleep deprivation on athletic performance. A meta-analysis of 22 randomised controlled trials involving 291 participants demonstrated that daytime naps lasting 30–60 min significantly enhanced cognitive and physical performance while reducing perceived fatigue [5]. Meta-analytic evidence indicates that the benefits of napping are optimised between 12:30 h and 16:50 h, with 14:00 h being the most effective time, and when the interval between nap awakening and performance testing exceeds one hour to minimise sleep inertia. Additionally, napping supports central nervous system recovery and improves hormone regulation, providing physiological mechanisms to counteract the effects of sleep loss [17,18]. These findings suggest that napping is a practical strategy for athletes facing sleep deprivation during intense training or competitions. Furthermore, napping serves as a critical tool for athletes competing in multi-event tournaments or back-to-back matches, where recovery time is limited, allowing for the rapid restoration of alertness without extended sleep periods [19].

The decision to opt for a 45 min nap was based on sleep architecture considerations and evidence from systematic reviews. This duration is intended to capture light-to-moderate NREM sleep while limiting the likelihood of deeper slow-wave sleep, which can increase post-nap grogginess and sleep inertia [20,21]. In line with prior work, naps were scheduled in the early afternoon, and performance testing was separated from awakening by at least one hour to reduce residual inertia effects and optimise readiness [5]. Although napping has been suggested as a countermeasure to sleep deprivation, its impact on mood states, anaerobic performance, and decision-making accuracy in martial arts has yet to be thoroughly quantified [16,22,23].

This study investigated the impact of a 45 min afternoon nap on mood, anaerobic performance, and decision-making in sleep-deprived elite Kung-Fu athletes. We hypothesised that napping would enhance cognitive function, particularly in terms of decision-making accuracy and reaction time, while improving mood by restoring vigour and reducing fatigue. Although we anticipated a modest effect on physical performance metrics, full recovery may have been limited by the effects of sleep deprivation. Consequently, this study aimed to quantify the extent to which a 45 min nap mitigates total sleep deprivation (TSD)-related impairments across the cognitive, mood, and anaerobic performance domains in elite Kung-Fu athletes.

## 2. Materials and Methods

This study examined the impact of a 45 min nap on mood, physical performance, and cognitive function in elite Kung-Fu athletes after 36 h of total sleep deprivation. Using a randomised, counterbalanced design, the investigation involved 24 male athletes and assessed four conditions (normal sleep (NS), without sleep deprivation (WSD) + nap, total sleep deprivation (TSD) (36 h awake), and TSD + nap) with 72 h recovery periods between each condition. Standardised controls were implemented for diet, circadian rhythm, and environmental factors.

### 2.1. Ethical Considerations

This study adhered to the Declaration of Helsinki [24] and was approved by the Ethics Committee of the High Institute of Sports and Physical Education of Sfax (CPP: 045/2019, 12 February 2022). All procedures complied with institutional guidelines for research involving human participants in sleep deprivation studies. Participants provided written informed consent after a comprehensive briefing session held one week before participation. The informed consent process provided detailed information about the study procedures, potential risks, benefits, and the right to withdraw without penalty. The specific risks disclosed included (1) temporary cognitive impairment during and immediately following sleep deprivation, (2) increased risk of accidents due to reduced alertness, (3) potential mood disturbances, and (4) possible disruption of sleep patterns requiring 3–5 days for complete recovery. Participants were advised to arrange transportation and avoid driving for 24 h after completing the study. Safety protocols during the 36 h sleep deprivation period included continuous medical supervision by qualified sports medicine personnel, with predetermined withdrawal criteria established to ensure participant safety. Emergency medical services were available throughout the study period. Data confidentiality was maintained through de-identification using unique participant codes, with the code key stored separately from the data files. All electronic data were stored on password-protected encrypted servers with restricted access, limited to the principal investigators.

### 2.2. Participants

Twenty-four elite male Kung-Fu athletes (age: 20.67 ± 1.76 years; height: 1.71 ± 0.04 m; BMI: 21.4 ± 2.1 kg/m^2^; training experience: 12.8 ± 2.1 years) participated in this study. Participants’ demographic and training characteristics are summarised in Table 1. These athletes were classified as competitive elites based on the criteria established by Swann, et al. [25] and competed at the national level with a minimum of 10 years of training experience. All participants engaged in low-intensity activities for approximately 10 h per week as part of their comprehensive training regimen. The inclusion criteria were as follows: (1) male sex; (2) competitive elite status; (3) age range of 18–25 years; (4) habitual sleep duration of 7–9 h; and (5) neutral chronotype classification according to the Morningness-Eveningness Questionnaire [26]. The exclusion criteria were as follows: (1) severe neuromuscular injuries; (2) diagnosed sleep disorders; (3) a history of shift work; (4) chronic medication use; (5) smoking or regular alcohol consumption; (6) cardiovascular disease; (7) psychiatric disorders; and (8) a history of substance abuse. Prescreening involved two sessions, each lasting 45–60 min, conducted at the sports physiology laboratory one week prior to the commencement of the study. Participants were given the Pittsburgh Sleep Quality Index (PSQI) (University of Pittsburgh, Pittsburgh, PA, USA) to evaluate sleep disorders and were instructed to maintain a 7-day sleep diary to verify their habitual sleep patterns [27]. A qualified sports medicine physician performed medical screenings using standardised health questionnaires and structured interviews. Recruitment was limited to male participants to control for potential sex differences in responses to sleep deprivation and napping while acknowledging the limitations of generalisability.

### 2.3. Experimental Design

In this study, a randomised, counterbalanced crossover design was utilised to evaluate the effects of sleep deprivation and napping interventions across four distinct test sessions, each separated by a 72 h washout period. The conditions were as follows: (1) no sleep deprivation (WSD), (2) WSD with napping, (3) total sleep deprivation (TSD), and (4) TSD with napping. The 72 h interval was selected to minimise carryover effects and was supported by prior crossover sleep studies; baseline recovery across outcomes was verified within the study before subsequent sessions [28,29]. The participants maintained habitual sleep durations of 7–9 h per night during the prescreening phase to establish their baseline sleep patterns [30]. To ensure standardisation throughout the experimental protocol, an 8 h sleep schedule (22:30–06:30) was enforced in the WSD condition to control for sleep duration as a potential confounding variable [31], with testing conducted at 17:30 h. The WSD with napping condition included a 45 min nap from 13:30 h to 14:15 h before the testing. The 36 h TSD condition was implemented under strict laboratory supervision with regular staff monitoring to ensure wakefulness and protocol adherence; participants were not permitted to lie down outside the scheduled nap periods [32]. In contrast, the TSD with napping condition included a post-deprivation nap of the same 45 min duration. Permissible activities during TSD were limited to low-intensity tasks (e.g., reading, quiet conversation, and board games; ≤1.5 METs), whereas vigorous physical activity, caffeine consumption, and exposure to electronic screens were prohibited.

**Figure 1:**
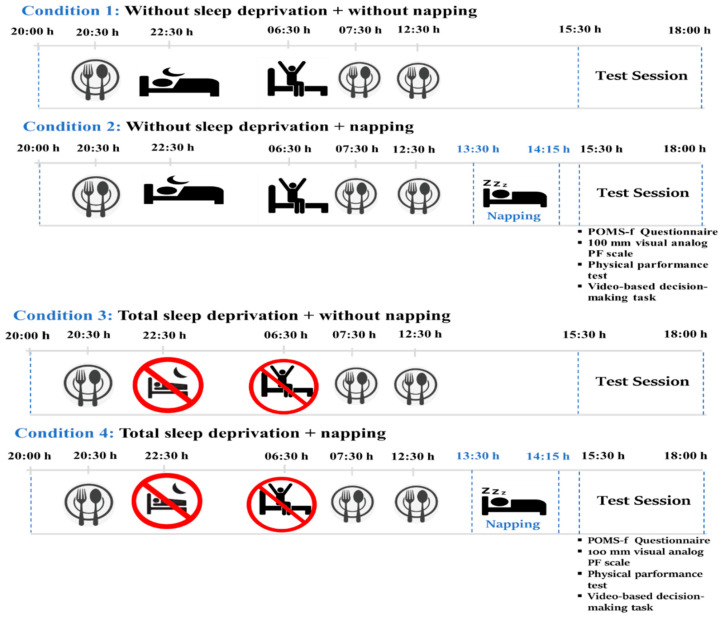
Schematic illustration of experimental design.

Testing was conducted under controlled conditions (21.1 ± 1.1 °C, 44.3 ± 7.6% humidity) and included a 10 min warm-up on a Monark cycloergometer (Ergomedic 874E Monark Exercise AB, SE-432 82 Varberg, Sweden). Profile of Mood States (POMS-f), physical tests (vertical/horizontal jumps, dynamometer strength, and medicine ball throw), and cognitive tasks (video-based decision-making and barrage test) [33,34]. Nutritional intake was standardised with meals matching the macronutrient composition (55% carbohydrate, 30% fat, and 15% protein). Compliance was ensured through consumption supervision and by using dietary recall questionnaires. Caffeine consumption was prohibited for 24 h before and during testing and was verified through questionnaires and supervision [35]. Participants gradually reduced their caffeine intake before testing [36]: 100% intake seven days prior, 50% five days prior, 25% three days prior, and 0% for 24 h prior to testing [37,38]. The 45 min nap was visually monitored. The participants were awakened by progressive light exposure, followed by 5 [39] min of light physical activity to minimise sleep inertia. Post-nap testing occurred 60–90 min after awakening for cognitive tests and 45 min for physical tests, ensuring adequate dissipation of any residual sleep inertia effects [40]. A schematic of this process is shown in Figure 1.

### 2.4. Dependent Measures

This study evaluated mood, perceptual response decision-making, and anaerobic performance as key dependent variables to determine the effects of sleep deprivation and 45 min of napping on Kung-fu athletes. These measures were chosen because of their proven sensitivity to sleep manipulation and relevance to athletic performance outcomes, as highlighted in recent systematic reviews and meta-analyses [1,2]. All instruments demonstrated acceptable psychometric properties for athletic populations, with test–retest reliability coefficients (ICC or r) ranging from 0.76 to 0.98 and established construct validity through correlations with objective performance measures (r = 0.58–0.78)

#### 2.4.1. Profile of Mood States

The Profile of Mood States Questionnaire in French (POMS-f) assesses six mood dimensions: anxiety, depression, anger, confusion, vigour, and fatigue [41]. The format comprises 65 adjectives rated on a 5-point Likert scale, ranging from 0 (‘not at all’) to 4 (‘extremely’), and requires approximately 5–10 min to complete. Total Mood Disturbance (TMD) was calculated as (Anxiety + Depression + Anger + Confusion + Fatigue) − vigour, with higher scores indicating a more negative mood. This instrument was selected because of its established use in sleep loss research and applicability in athletic populations [19,42,43,44].

#### 2.4.2. Perceptual Responses

Perceptual responses were assessed using a 100 mm horizontal visual analogue scale (VAS) for concentration, attention, fatigue, and sleep quality [45,46]. The VAS format was selected for its sensitivity to transient state changes, minimal learning effects, and established validity in sleep deprivation research [45,46]. The VAS format was selected for its sensitivity to transient state changes, minimal learning effects, and established validity in sleep deprivation research [45,46]. Participants marked a single point on each scale to reflect their current state. The scores were determined by measuring the distance (mm) from the left anchor to each mark. Each scale required approximately 30 s to complete, with endpoints defined as follows: concentration (0 = ‘Cannot concentrate at all’ to 100 = ‘Completely focused’) [47]; attention (0 = ‘Not at all attentive’ to 100 = ‘Extremely attentive’); fatigue (0 = ‘Not at all tired’ to 100 = ‘Extremely tired’); and sleep quality (0 = ‘Very poor’ to 100 = ‘Excellent’).

- Concentration

The Visual Analogue Scale (VAS) for concentration was used to assess perceived attention maintenance, employing a 100 mm horizontal line format. Participants marked their current concentration level on this scale, which ranged from “0 = Cannot concentrate at all” to “100 = Completely focused” [47]. The administration of this instrument required approximately 30 s. The VAS was selected for its proven sensitivity to the effects of sleep deprivation on subjective attention states and its suitability for repeated measurements without causing learning effects [48]. The test–retest reliability of the VAS was demonstrated by intraclass correlation coefficients (ICC = 0.78–0.85) over 24 h intervals in athletic populations [49]. Convergent validity was supported by moderate-to-strong correlations (r = 0.62–0.74, *p* < 0.001) with objective performance measures, confirming that subjective concentration ratings were meaningfully aligned with cognitive performance outcomes. Scoring involved measuring the distance from the left anchor point in millimetres, with higher scores indicating better-perceived concentration. The VAS format is preferred over Likert scales because of its superior sensitivity to small changes in subjective states [50].

- Attention

The visual analogue scale (VAS) for attention is a tool designed to evaluate subjective vigilance and the ability to maintain alertness. It measures participants’ perceived momentary attentional states using a 100 mm horizontal line, where participants mark their current level of attention, from “0 = Not at all attentive” to “100 = Extremely attentive.” The administration of this scale required approximately 30 s. The attention VAS was selected for its proven capability to detect rapid changes in attentional state after interventions, such as napping, and its established sensitivity to sleep manipulation in athletic populations [50]. The VAS demonstrates strong psychometric properties, including test–retest reliability (ICC = 0.76–0.82) over 24 h intervals [51]. Convergent validity was shown by moderate-to-strong correlations (r = 0.58–0.71, *p* < 0.001) with objective measures of attention. Its validity is further supported by its sensitivity to sleep deprivation, which is consistent with the established findings on the cognitive effects of sleep loss [52]. Scoring involved measuring the distance in millimetres from the left anchor point to the participant’s mark, with higher scores indicating better perceived attention. This instrument was favoured over discrete rating scales because of its superior sensitivity to small changes in subjective attentional states and its reduced ceiling/floor effects in repeated-measures designs [53].

- Fatigue

The Visual Analogue Scale (VAS) for fatigue is a tool designed to measure subjective levels of physical and mental exhaustion at a specific moment. This scale uses a 100 mm horizontal line, where participants mark their current fatigue level, ranging from “0 = Not at all tired” to “100 = Extremely tired,” with the process lasting approximately 30 s. The fatigue VAS was chosen for its proven sensitivity to temporary fatigue states caused by sleep deprivation, ease of repeated use without learning effects, and its established validity in athletic populations [54]. The test–retest reliability of the fatigue VAS has been confirmed with intraclass correlation coefficients (ICC) ranging from 0.82 to 0.89 over 24–48 h intervals in similar populations [51]. Internal consistency in repeated-measures designs showed good reliability (r = 0.76–0.84) for detecting changes related to interventions [55]. Convergent validity was supported by moderate-to-strong correlations (r = 0.64–0.78, *p* < 0.001) with objective performance measures, including physical tasks (medicine ball throw, vertical jump) and cognitive accuracy measures, confirming that subjective fatigue ratings meaningfully corresponded to actual performance decline [50]. Scoring involved measuring the distance from the left anchor point (“0 = Not at all tired”) to the participant’s mark in millimetres, with higher scores indicating greater perceived fatigue. The VAS format is preferred over discrete rating scales because of its superior sensitivity to small changes in fatigue states and reduced response bias in repeated measures designs [53].

- Sleep Quality

Sleep quality was evaluated using a 100 mm horizontal visual analogue scale (VAS), where participants rated their perceived sleep sufficiency from “0 = Very poor sleep quality” to “100 = Excellent sleep quality” [56,57]. Each assessment took approximately 30 s. The VAS was chosen over objective measures, such as actigraphy and polysomnography, to maintain consistency with other subjective metrics in the study design, as the primary focus was on performance outcomes rather than a detailed analysis of sleep architecture [51]. Although the VAS has demonstrated sensitivity to acute changes in perceived sleep quality across various contexts, it is important to note that this tool may have limited sensitivity in detecting subtle differences between experimental conditions, particularly those related to brief napping interventions [58]. The test–retest reliability of the sleep quality VAS has been established, with correlation coefficients ranging from r = 0.72–0.84 over 24–48 h intervals [51]. The instrument showed adequate construct validity through its alignment with the expected effects of sleep deprivation and moderate correlations (r = 0.58–0.67) with objective sleep measures when available [59]. Scoring involved measuring the distance from the left anchor point to the participant’s mark in millimetres, with higher scores indicating better perceived sleep quality. Standardised administration protocols were implemented across all assessments to ensure consistency and reproducibility [58].

#### 2.4.3. Decision-Making Skills

Decision-making was assessed using the Combat Tactical Decision-Making Task (CTDMT) [60], in which participants viewed 30 paused Kung-Fu combat scenarios (4–10 s) at critical decision points and selected the most effective action (upper strike, middle strike, lower strike, or defensive repositioning) [61,62]. The scenarios were projected onto a 4 m × 3 m wall at a viewing distance of 3 m [63]. Correct responses were defined by a panel of national-level kung-fu coaches [64]. Participants responded within 3 s using the keyboard keys (Q/W/E/R), and the accuracy (% correct) and reaction time (stimulus onset to keypress) were logged automatically [65]. A panel of five national-level Kung-Fu coaches (mean experience = 18.3 ± 4.2 years) determined the correct responses, demonstrating excellent interrater reliability using a two-way random-effects model with absolute agreement (ICC [2, 1] = 0.88, 95% CI [0.82–0.93]) [64]. This methodology provides a precise measurement of decision-making under high-pressure athletic conditions, with established psychometric properties suitable for research applications [66].

#### 2.4.4. Barrage Test

The Barrage Test was used to evaluate sustained attention, concentration, and perceptual speed [67]. This task required participants to work on a sheet containing 1000 symbols (squares with lines of various orientations) arranged in 40 rows. Participants were required to identify and mark symbols that matched two predefined target models among distractors within a 10 min timeframe. Performance metrics were recorded at 2-, 5-, 8-, and 10-min intervals to assess attention stability and potential fatigue effects. A composite Barrage Score was calculated using the following formula: (number of correct responses-number of errors)/total time × 100, with higher scores indicating superior attentional performance and processing efficiency. The test was conducted in a quiet environment with standardised instructions, and the participants were seated at individual desks under consistent lighting conditions (500–800 lux).

#### 2.4.5. Anaerobic Performances

Anaerobic performance was assessed via vertical jump (OptoJump Next, Microgate S.r.l., Bolzano, Italy), horizontal jump, seated 2 kg medicine ball throw, and isometric back/leg strength (Takei 5402 Back-D dynamometer, Takei Scientific Instruments Co., Ltd., Niigata, Japan) [68]. After a 10 min warm-up [69], the tests were administered in a standardised order with a 5 min recovery period between modalities [70,71] Vertical jump: This involved two maximal trials (60 s rest) using ~90° knee flexion with arm swing. Horizontal jump: This involved two trials (60 s rest) using a ~120° knee angle; distance was recorded to the closest heel with ≥2 s balance required. Medicine ball throw: This involved participants being seated against a wall (legs extended) and throwing the ball at ~45°, with the best of two attempts recorded (60 s rest) [68,72]. Isometric strength: A dynamometer calibrated before each session; back strength was measured at 30° lumbar flexion (standing) and leg strength at 135° knee flexion (seated), with three trials per muscle group (60 s rest), and the highest value was retained [73,74]. Testing was conducted under controlled conditions (20–22 °C; 45–55% humidity) and at consistent times (14:00–17:00 h) [75,76,77].

### 2.5. Statistical Analysis

Data are presented as the mean ± standard deviation (M ± SD) values. A 2 × 2 repeated-measures analysis of variance (ANOVA) was conducted to evaluate both within-group and between-group differences, with sleep conditions (deprivation vs. no deprivation) and nap conditions (presence vs. absence) as the within-subject factors. The assumptions for ANOVA were assessed as follows: normality of residuals was evaluated using the Shapiro–Wilk test [78], outliers (defined as values exceeding 2.5 standard deviations) were identified using studentized residuals [79], and sphericity was tested using Mauchly’s test, applying the Greenhouse–Geisser correction when sphericity was violated (*p* < 0.05) [80]. Statistical significance was determined using a two-way repeated-measures ANOVA. For significant main effects and interactions, planned comparisons were conducted using Bonferroni-corrected post hoc tests for pairwise comparisons at each factor level. In factorial designs, significant interactions are followed by simple main effect analyses to examine the effects of one factor on the other’s level [81]. For all significant effects, we reported the absolute mean differences between conditions, 95% confidence intervals, effect sizes (partial η^2^), and post hoc test results with appropriate corrections for multiple comparisons. To control for family wise error rates across multiple outcome domains, we implemented the Benjamini–Hochberg False Discovery Rate (FDR) procedure with *p* = 0.05, which was applied separately within each outcome family [82]. The tests were grouped into distinct families based on the following outcome domains: (1) mood states (POMS-f dimensions), (2) perceptual responses (VAS scores for concentration, attention, fatigue, and sleep quality), (3) decision-making metrics (accuracy and reaction time), and (4) cognitive performance (barrage test metrics). This two-tiered approach used the Bonferroni correction for pairwise comparisons within each ANOVA, followed by the FDR correction across outcome families to control the overall Type I error rate while maintaining adequate statistical power. This approach was chosen over an experiment-wide Bonferroni correction to balance Type I error control while maintaining reasonable statistical power, which is particularly important given the sample size and the multiple outcome domains [83]. For transparency, both uncorrected and FDR-adjusted *p*-values are reported in the Results Section, with statistical significance determined based on FDR-adjusted values (*p* < 0.05). This methodological decision was guided by recommendations for repeated-measures designs with multiple dependent variables [84], where domain-specific error control is more suitable than experiment-wide corrections, which may be overly punitive and increase Type II error rates. Effect sizes were expressed as partial eta squared (ηp^2^) for ANOVA effects and Cohen’s d for pairwise comparisons, providing insights into both the statistical and practical significance. A priori power analysis, conducted using G*Power (Version 3.1.9.7; Heinrich-Heine-Universität Düsseldorf, Düsseldorf, Germany), indicated that a sample size of 24 participants would be sufficient to detect medium-to-large effects (Cohen’s f = 0.30) with α = 0.05, and a power of 0.80, for the primary outcome measures, based on effect sizes observed in similar sleep deprivation studies [85]. Because a counterbalanced repeated-measures crossover design was employed, each participant served as their own control across all conditions; therefore, randomisation did not reduce the effective sample size for statistical power. Cohen’s d values were interpreted according to established conventions: small (d = 0.20), medium (d = 0.50), and large (d = 0.80) [85]. Partial eta-squared values were interpreted as small (ηp^2^ = 0.01), medium (ηp^2^ = 0.06), or large (ηp^2^ = 0.14) [85]. All statistical analyses were performed using IBM SPSS (Version 26; IBM Corp., Armonk, NY, USA).

## 3. Results

### 3.1. Effects on Psychological Parameters

#### 3.1.1. Profile of Mood States (POMS)

##### Effects of Sleep Deprivation on Mood States

A two-way repeated measures ANOVA was conducted to investigate the impact of total sleep deprivation (TSD) on mood states in 24 elite kung-fu athletes. Sleep deprivation significantly influenced five mood dimensions.

Depression exhibited a significant main effect on sleep condition [F(1, 23) = 29.99, *p* < 0.001, ηp^2^ = 0.56], with lower scores in the TSD − N condition (4.00 ± 3.75) than in the WSD − NAP condition ((9.76 ± 8.07), MD = 5.76, 95% CI [2.89, 8.63], *p* < 0.001, d = 0.87, 95% CI [0.29, 1.45]). Tension also showed a significant main effect [F(1, 23) = 15.32, *p* = 0.001, ηp^2^ = 0.39], with higher scores in the TSD − N condition (15.60 ± 2.10) than in the WSD − NAP condition ((9.36 ± 4.99), MD = 6.24, 95% CI [2.76, 9.72], *p* = 0.001, d = 1.57, 95% CI [0.89, 2.25]). Confusion demonstrated a significant main effect [F(1, 23) = 88.11, *p* < 0.001, ηp^2^ = 0.79], with increased scores in the TSD − N condition (14.32 ± 1.89) compared to the WSD − NAP condition)(7.00 ± 3.52), MD = 7.32, 95% CI [5.87, 8.77], *p* < 0.001, d = 2.55, 95% CI [1.74, 3.36]).

Vigour showed a significant main effect [F(1, 23) = 66.58, *p* < 0.001, ηp^2^ = 0.97], with notably lower scores in the TSD − N condition (9.48 ± 2.24) than in the WSD − NAP condition ((21.20 ± 2.69), MD = 11.72, 95% CI [9.74, 13.70], *p* < 0.001, d = 4.73, 95% CI [3.65, 5.81]). Fatigue also exhibited a significant main effect [F(1, 23) = 90.31, *p* < 0.001, ηp^2^ = 0.79], with higher scores in the TSD − N condition (17.20 ± 2.74) than in the WSD − NAP condition ((5.24 ± 4.00), MD = 11.96, 95% CI [9.51, 14.41], *p* < 0.001, d = 3.46, 95% CI [2.58, 4.34]).

Anger did not display a significant main effect of sleep deprivation [F(1, 23) = 0.48, *p* = 0.50, ηp^2^ = 0.02], with similar scores between the TSD − N condition (15.8 ± 2.90) and WSD − NAP condition (12.36 ± 7.58).

##### Effects of Napping on Mood States

Napping significantly impacted five of the six mood dimensions. Anger exhibited a notable main effect of napping [F(1, 23) = 23.14, *p* < 0.001, ηp^2^ = 0.49], with scores being lower in the nap condition than in the no-nap condition. Tension also showed a significant main effect [F(1, 23) = 19.15, *p* < 0.001, ηp^2^ = 0.44], with scores decreasing after the nap. Confusion presented a significant main effect [F(1, 23) = 17.24, *p* < 0.001, ηp^2^ = 0.42], with lower scores in the nap condition. Vigour displayed a significant, albeit smaller, main effect [F(1, 23) = 5.95, *p* = 0.022, ηp^2^ = 0.20], with slightly higher scores in the nap condition. Fatigue showed the most substantial main effect [F(1, 23) = 114.41, *p* < 0.001, ηp^2^ = 0.83], with scores significantly lower after napping than after not napping. However, depression did not exhibit a significant main effect on napping [F(1, 23) = 0.10, *p* = 0.76, ηp^2^ = 0.004].

##### Sleep–Nap Interaction Effects on Mood States

Significant interaction effects between sleep conditions and napping were identified across four mood dimensions. The most pronounced interaction effect was observed for fatigue [F(1, 23) = 64.03, *p* < 0.001, ηp^2^ = 0.73], followed by tension [F(1, 23) = 26.29, *p* < 0.001, ηp^2^ = 0.52], confusion [F(1, 23) = 25.51, *p* < 0.001, ηp^2^ = 0.52], and anger [F(1, 23) = 9.37, *p* = 0.005, ηp^2^ = 0.28]. For these significant interactions, the mean differences between the TSD − N and TSD + NAP conditions were notably greater than those between the WSD conditions. Specifically, the differences in anger were 7.36 points [95% CI [5.84, 8.88]] for the TSD conditions compared to 2.12 points [95% CI [0.37, 3.87]] for the WSD condition. Tension differences were 6.28 points [95% CI [4.76, 7.80]] for TSD conditions versus 0.04 points [95% CI [−1.48, 1.56]] for WSD conditions. The confusion differences were 5.16 points [95% CI [3.61, 6.71]] for TSD conditions compared to 0.32 points [95% CI [−1.23, 1.87]] for WSD conditions. Fatigue differences were 10.52 points [95% CI [8.74, 12.30]] for TSD conditions versus 0.72 points [95% CI [−1.06, 2.50]] for WSD conditions. No significant interaction effects were detected for depression [F(1, 23) = 0.57, *p* = 0.460, ηp^2^ = 0.02] or vigour [F(1, 23) = 0.001, *p* = 0.97, ηp^2^ = 0.000] (Table 2).

#### 3.1.2. Subjective Perceptions of Sleepiness and Recovery

##### Effects of Sleep Deprivation on Perceived Sleepiness

A repeated-measures ANOVA revealed that the TSD condition significantly impacted the five subjective perception measures. Notably, sleep quality showed a significant main effect [F(1, 23) = 474.04, *p* < 0.001, ηp^2^ = 0.95], with scores of 1.00 ± 0.00 in the TSD condition compared with 4.00 ± 0.78 in the WSD condition [MD = 3.00, 95% CI [2.65, 3.35], *p* < 0.001, d = 5.41, 95% CI [4.01, 6.81]]. Fatigue also exhibited a significant main effect [F(1, 23) = 975.59, *p* < 0.001, ηp^2^ = 0.98], with significantly higher scores in the TSD condition (87.80 ± 10.81) than in the WSD condition (12.00 ± 7.91) [MD = 75.80; 95% CI = [70.52, 81.08]; *p* < 0.001; d = 7.85; 95% CI = [5.89, 9.81]].

Additionally, sleep quantity demonstrated a significant main effect [F(1, 23) = 411.89, *p* < 0.001, ηp^2^ = 0.95], with a lower perceived quantity in the TSD condition (4.15 ± 1.73) than in the WSD condition (7.90 ± 1.75) [MD = 3.75, 95% CI [3.01, 4.49], *p* < 0.001, d = 2.16, 95% CI [1.44, 2.88]]. Concentration showed a significant main effect [F(1, 23) = 641.61, *p* < 0.001, ηp^2^ = 0.96], with considerably lower scores in the TSD group (25.80 ± 9.45) than in the WSD group (87.40 ± 11.55) [MD = 61.60, 95% CI [56.17, 67.03], *p* < 0.001, d = 5.88, 95% CI [4.32, 7.44]]. Attention also displayed a significant main effect [F(1, 23) = 951.78, *p* < 0.001, ηp^2^ = 0.98], with lower scores in the TSD group (24.80 ± 7.63) than in the WSD group (87.30 ± 12.16) [MD = 62.50, 95% CI [58.42, 66.58], *p* < 0.001, d = 6.16, 95% CI [4.57, 7.75]].

##### Effects of Napping on Perceived Recovery

Napping significantly influenced four of the five subjective perception measures in this study. Fatigue showed a notable main effect [F(1, 23) = 30.06, *p* < 0.001, ηp^2^ = 0.56], with lower scores in the nap condition (37.30 ± 8.54) than in the no-nap condition (49.90 ± 13.78) [MD = 12.60, 95% CI [8.12, 17.08], *p* < 0.001, d = 1.10, 95% CI [0.52, 1.68]]. Sleep quantity also displayed a significant main effect [F(1, 23) = 229.64, *p* < 0.001, ηp^2^ = 0.95], with a lower perceived quantity in the nap condition (3.89 ± 1.75) than in the no-nap condition (8.15 ± 1.46) [MD = 4.26, 95% CI [3.70, 4.82], *p* < 0.001, d = 2.64, 95% CI [1.85, 3.43]].

Concentration exhibited a significant main effect [F(1, 23) = 35.26, *p* < 0.001, ηp^2^ = 0.60], with improved scores in the nap condition (61.30 ± 11.13) compared to the no-nap condition (51.90 ± 9.45) [MD = 9.40, 95% CI [6.21, 12.59], *p* = 0.002, d = 0.89, 95% CI [0.32, 1.46]]. Attention also showed a significant main effect [F(1, 23) = 20.87, *p* < 0.001, ηp^2^ = 0.47], with higher scores in the nap condition (60.40 ± 10.77) than in the no-nap condition (51.70 ± 7.63) [MD = 8.70, 95% CI [4.65, 12.75], *p* = 0.008, d = 0.92, 95% CI [0.34, 1.50]].

In contrast, sleep quality did not show a significant main effect of napping [F(1, 23) = 0.38, *p* = 0.543, ηp^2^ = 0.02], indicating that napping did not significantly alter the perceived sleep quality.

##### Sleep–Nap Interaction Effects on Subjective Measures

Significant interaction effects between sleep conditions and napping were observed for all four measures. Fatigue exhibited a notable interaction [F(1, 23) = 29.14, *p* < 0.001, ηp^2^ = 0.55], with napping yielding more pronounced benefits under sleep deprivation conditions. The mean difference between the TSD conditions (no nap vs. nap) was 25.20 points [95% CI [20.44, 29.96]], whereas the difference between the WSD conditions was 2.40 points [95% CI [0.96, 3.84]].

Sleep quantity also showed a significant interaction [F(1, 23) = 88.29, *p* < 0.001, ηp^2^ = 0.89], with mean differences of 8.30 points [95% CI [7.55, 9.05]] between TSD conditions, compared to 0.30 points [95% CI [0.12, 0.48]] between WSD conditions. Concentration demonstrated a significant interaction [F(1, 23) = 14.37, *p* = 0.001, ηp^2^ = 0.37], with differences of 27.80 points [95% CI [22.83, 32.77]] between TSD conditions and 4.20 points [95% CI [2.10, 6.30]] between WSD conditions. Attention also showed a significant interaction [F(1, 23) = 18.04, *p* < 0.001, ηp^2^ = 0.43], with differences of 26.60 points [95% CI [21.78, 31.42]] between the TSD conditions compared to 3.40 points [95% CI [1.70, 5.10]] between the WSD conditions.

The effect sizes for the TSD condition differences were exceptionally large: fatigue (d = 2.14, 95% CI [1.41, 2.87]), sleep quantity (d = 6.78, 95% CI [4.98, 8.58]), concentration (d = 2.71, 95% CI [1.93, 3.49]), and attention (d = 3.01, 95% CI [2.18, 3.84]).

Sleep quality did not exhibit a significant interaction effect [F(1, 23) = 2.76, *p* = 0.110, ηp^2^ = 0.10] (Table 3).

### 3.2. Effects on Cognitive Function

#### 3.2.1. Decision-Making Performance

##### Effects of Sleep Deprivation on Decision-Making

A repeated-measures ANOVA was performed to assess the impact of TSD on decision accuracy (DA) and decision time (DT) among the 24 elite kung-fu athletes. Sleep deprivation had a significant effect on decision accuracy [F(1, 23) = 131.67, *p* < 0.001, ηp^2^ = 0.85] and decision time [F(1, 23) = 88.06, *p* < 0.001, ηp^2^ = 0.79]. The decision time was notably longer in the TSD condition (0.92 ± 0.13 s) than in the WSD condition (0.58 ± 0.08 s) (MD = 0.34, 95% CI [0.26, 0.42]; *p* < 0.001; d = 3.14; 95% CI [2.32, 3.96]). Similarly, decision accuracy was significantly lower in the TSD condition (18.44 ± 2.24) than in the WSD condition (24.00 ± 2.16) (MD = 5.56, 95% CI [4.32, 6.80], *p* < 0.001, d = 2.52, 95% CI [1.76, 3.28]).

##### Effects of Napping on Decision-Making

Napping had a significant impact on both decision time [F(1, 23) = 42.99, *p* < 0.001, ηp^2^ = 0.64] and accuracy [F(1, 23) = 26.67, *p* < 0.001, ηp^2^ = 0.53]. In the nap condition, the decision time was notably shorter (0.69 ± 0.09 s) than that in the no-nap condition (0.75 ± 0.13 s), with MD = 0.06, 95% CI [0.02, 0.10], *p* = 0.002, d = 0.53, 95% CI [0.12, 0.94]. Similarly, decision accuracy was significantly higher in the nap condition (22.22 ± 2.23) than in the no-nap condition (21.22 ± 2.24), with MD = 1.00, 95% CI [0.26, 1.74], *p* = 0.009, d = 0.45, 95% CI [0.04, 0.86].

##### Sleep–Nap Interaction Effects on Decision-Making

Significant interaction effects between sleep conditions and napping were observed for both decision accuracy [F(1, 23) = 50.36, *p* < 0.001, ηp^2^ = 0.68], and decision time [F(1, 23) = 45.78, *p* < 0.001, ηp^2^ = 0.67]. In terms of decision accuracy, napping led to notably greater improvements under sleep deprivation than under the well-rested condition. The mean difference between the TSD − N and TSD + NAP conditions was 2.60 points [95% CI [1.94, 3.26]], indicating a 14.1% improvement, whereas the difference between the WSD − NAP and WSD + NAP conditions was −0.60 points [95% CI [−1.06, −0.14]], reflecting a 2.5% decrease. Regarding decision time, the interaction showed that napping had a more pronounced positive effect on sleep deprivation than on sleep deprivation. The mean difference between the TSD − N and TSD + NAP conditions was 0.15 s [95% CI [0.11, 0.19]], while the difference between the WSD − NAP and WSD + NAP conditions was 0.01 s [95% CI [0.00, 0.02]]. The effect sizes for the TSD condition differences were substantial: decision accuracy (d = 1.17, 95% CI [0.59, 1.75]) and decision time (d = 1.32, 95% CI [0.72, 1.92]). Conversely, the effect sizes for the WSD condition differences were small: decision accuracy (d = −0.28, 95% CI [−0.85, 0.29]) and decision time (d = 0.13, 95% CI [−0.44, 0.70]). Post hoc Bonferroni tests confirmed significant improvements in both decision accuracy and decision time for the TSD condition (*p* < 0.001 for both), but not for the WSD condition (decision accuracy, *p* = 0.13; decision time, *p* = 0.33) (Table 4).

#### 3.2.2. Sustained Attention Performance (Barrage Test)

##### Effects of Sleep Deprivation on Sustained Attention

A repeated-measures ANOVA indicated that TSD significantly impaired performance in the barrage test [F(1, 23) = 69.70, *p* < 0.001, ηp^2^ = 0.74] among the 24 elite Kung-Fu athletes. The barrage test scores were notably lower under the TSD condition (53.14 ± 11.32) than under the WSD condition (63.80 ± 9.82), with a mean difference of 10.66 (95% CI [8.09, 13.23], *p* < 0.001, d = 0.98, 95% CI [0.40, 1.56]).

##### Effects of Napping on Sustained Attention

Napping did not have a significant main effect on barrage test performance [F(1, 23) = 0.82, *p* = 0.374, ηp^2^ = 0.03]. There were no significant differences in the barrage test scores between the nap condition (59.48 ± 12.18) and the no-nap condition ((57.48 ± 13.70), MD = 2.00, 95% CI [−2.47, 6.47], *p* = 0.374, d = 0.15, 95% CI [−0.42, 0.72]).

##### Sleep–Nap Interaction Effects on Sustained Attention

A significant interaction between sleep condition and napping was observed in barrage test performance [F(1, 23) = 11.97, *p* = 0.002, ηp^2^ = 0.33]. This interaction highlights that the effects of napping vary depending on sleep conditions. Specifically, the mean difference in barrage test scores between the TSD − N and TSD + NAP conditions was 7.08 points [95% CI [4.95, 9.21]], whereas the difference between the WSD − NAP and WSD + NAP conditions was −3.08 points [95% CI [−5.26, −0.90]]. Post hoc Bonferroni tests indicated that participants in the TSD − N condition had significantly lower barrage test scores (49.60 ± 11.32) than those in the TSD + NAP condition (56.68 ± 13.70), with MD = 7.08, 95% CI [4.95, 9.21], *p* = 0.008, d = 0.56, 95% CI [0.14, 0.98]. Conversely, scores in the WSD − NAP condition (65.36 ± 9.82) were significantly higher than those in the WSD + NAP condition (62.28 ± 12.18), with MD = 3.08, 95% CI [0.90, 5.26], *p* = 0.043, d = 0.28, 95% CI [−0.29, 0.85] (Table 5).

### 3.3. Effects on Physical Performance

#### 3.3.1. Effects of Sleep Deprivation on Physical Performance Measures

Repeated-measures ANOVA indicated that TSD had a significant impact on all physical performance parameters in the 24 elite kung-fu athletes. For horizontal jump performance, there was a notable main effect [F(1, 23) = 12.63, *p* = 0.002, ηp^2^ = 0.35], with longer distances recorded in the TSD condition (2.12 ± 0.21 m) than in the WSD condition)(2.10 ± 0.21 m), MD = 0.02, 95% CI [0.01, 0.03], *p* = 0.002, d = 0.10, 95% CI [−0.47, 0.67]). The vertical jump also showed a significant main effect [F(1, 23) = 35.68, *p* < 0.001, ηp^2^ = 0.56], with lower heights in the TSD condition (33.23 ± 6.03 cm) than in the WSD condition ((36.80 ± 6.47 cm), MD = 3.57, 95% CI [2.36, 4.78], *p* < 0.001, d = 0.57, 95% CI [0.16, 0.98]).

Isometric leg muscle strength exhibited a significant main effect [F(1, 23) = 10.59, *p* = 0.003, ηp^2^ = 0.31], with reduced strength in the TSD condition (110.10 ± 19.79 kg) compared to the WSD condition (121.10 ± 27.88 kg) (MD = 11.00, 95% CI [4.23, 17.77], *p* = 0.003, d = 0.45, 95% CI [0.04, 0.86]). Similarly, isometric back muscle strength showed a significant main effect [F(1, 23) = 5.95, *p* = 0.022, ηp^2^ = 0.20], with decreased strength in the TSD condition (128.40 ± 30.01 kg) compared to the WSD condition)(140.00 ± 38.08 kg), MD = 11.60, 95% CI [1.84, 21.36], *p* = 0.022, d = 0.34, 95% CI [−0.23, 0.91]). The seated medicine ball throw also demonstrated a significant main effect [F(1, 23) = 16.52, *p* < 0.001, ηp^2^ = 0.41], with shorter distances in the TSD condition (4.40 ± 0.69 m) than in the WSD condition)(4.47 ± 0.70 m), MD = 0.07, 95% CI [0.03, 0.11], *p* < 0.001, d = 0.10, 95% CI [−0.47, 0.67]).

#### 3.3.2. Effects of Napping on Physical Performance Measures

Napping significantly affected two of the five physical performance measures. Seated medicine ball throws demonstrated a significant main effect [F(1, 23) = 103.06, *p* < 0.001, ηp^2^ = 0.81], with improved performance in the nap conditions (4.44 ± 0.69 m) compared to the no-nap conditions (4.21 ± 0.66 m), MD = 0.23, 95% CI [0.18, 0.28], *p* < 0.001, d = 0.34, 95% CI [−0.23, 0.91]. Horizontal jump showed a significant main effect [F(1, 23) = 76.25, *p* < 0.001, ηp^2^ = 0.76], with greater distances in nap conditions (2.10 ± 0.21 m) compared to no-nap conditions (2.01 ± 0.18 m), MD = 0.09, 95% CI [0.07, 0.11], *p* < 0.001, d = 0.47, 95% CI [0.06, 0.88].

No significant main effects of napping were observed for vertical jump [F(1, 23) = 2.84, *p* = 0.105, ηp^2^ = 0.11], isometric leg muscle strength [F(1, 23) = 1.92, *p* = 0.18, ηp^2^ = 0.078], or isometric back muscle strength [F(1, 23) = 0.68, *p* = 0.418, ηp^2^ = 0.029].

#### 3.3.3. Sleep–Nap Interaction Effects on Physical Performance

Significant interaction effects between sleep conditions and napping were identified for four of the five physical performance measures. Specifically, the horizontal jump showed a significant interaction [F(1, 23) = 56.80, *p* < 0.001, ηp^2^ = 0.70], similar to the vertical jump [F(1, 23) = 5.68, *p* = 0.025, ηp^2^ = 0.191], isometric back muscle strength [F(1, 23) = 5.95, *p* = 0.023, ηp^2^ = 0.20], and seated medicine ball throw [F(1, 23) = 31.50, *p* < 0.001, ηp^2^ = 0.57]. These interactions indicate that napping leads to greater performance enhancement under sleep deprivation conditions than under well-rested conditions. For the horizontal jump, the mean difference between the TSD − N and TSD + NAP conditions was 0.10 m [95% CI [0.08, 0.12]], whereas the difference between the WSD − NAP and WSD + NAP conditions was 0.02 m [95% CI [0.01, 0.03]]. In the case of the vertical jump, the differences were 0.79 cm [95% confidence interval CI [0.42, 1.16]] versus 0.35 cm [95% CI [0.19, 0.51]]. For isometric back muscle strength, the differences were 7.80 kg [95% CI [5.12, 10.48]] versus 2.70 kg [95% CI [1.34, 4.06]]. Regarding the seated medicine ball throw, the differences were 0.36 m [95% CI [0.29, 0.43]] versus 0.08 m [95% CI [0.05, 0.11]]. Post hoc Bonferroni tests demonstrated that horizontal jump performance was significantly superior in the WSD + NAP condition (2.10 ± 0.21 m) than in the TSD + NAP condition ((1.92 ± 0.18 m), MD = 0.18, 95% CI [0.12, 0.24], *p* < 0.001, d = 0.90, 95% CI [0.31, 1.49]). Similarly, vertical jump performance was significantly higher in the WSD + NAP condition (36.45 ± 5.94 cm) than in the TSD + NAP condition (34.02 ± 6.14 cm) (MD = 2.43, 95% CI [0.31, 4.55], *p* = 0.025, d = 0.40, 95% CI [−0.17, 0.97]). Seated medicine ball throw performance was also significantly greater in the WSD + NAP condition (4.39 ± 0.66 m) than in the TSD + NAP condition (4.04 ± 0.63 m) (MD = 0.35, 95% CI [0.23, 0.47], *p* < 0.001, d = 0.54, 95% CI [−0.03, 1.11]). No significant interaction effect was observed for isometric leg muscle strength [F(1, 23) = 1.45, *p* = 0.24, ηp^2^ = 0.06] (Table 6).

## 4. Discussion

### 4.1. Research Problem and Objectives

This study investigated the effects of a 45 min nap on subjective experience, physical performance, and cognitive function in elite Kung-Fu athletes after total sleep deprivation (TSD). This study addresses a critical gap in the combat sports science literature by examining napping as a targeted recovery intervention for athletes who must integrate explosive physical performance with rapid tactical decision-making under competitive pressure.

### 4.2. Summary of Key Findings

The primary findings of this study indicate that napping leads to varied recovery patterns across performance domains following TSD exposure. Cognitive performance showed significant recovery, with decision-making accuracy improving by 14.1% (Cohen’s d = 1.17) and a 16.1% reduction in reaction time after napping in sleep-deprived conditions. Mood states exhibited notable restoration, particularly vigour (53.8% recovery), along with reduced fatigue, tension, and confusion. Physical performance demonstrated modest yet significant improvements, with a 2.4% increase in vertical jump and a slight enhancement in seated medicine ball throw performance. Crucially, the study uncovered significant sleep × nap interaction effects across all measured domains, indicating that the benefits of napping were considerably greater under sleep-deprived conditions than when well rested. This pattern suggests that tapping primarily serves as a restorative rather than a performance-enhancing intervention.

### 4.3. Interpretation of Results

The distinct recovery patterns observed in the cognitive, mood, and physical domains highlight the unique neurophysiological mechanisms affected by sleep loss, which are subsequently restored by napping. Cognitive performance improvements align with established sleep science principles, where brief naps preferentially rejuvenate the prefrontal cortex, which is crucial for executive control, attention regulation, and decision-making [86]. These findings suggest that cognitive systems exhibit greater neuroplasticity and faster recovery kinetics than physical performance systems [40,87,88]. This disparity may be due to the differing metabolic demands and recovery timelines required for neural and muscular restoration. Significant mood enhancement, particularly vigour restoration, indicates that strategic napping effectively counters the emotional dysregulation often observed after sleep deprivation. This mood restoration likely reflects the rapid normalisation of neurotransmitter systems, especially dopamine and serotonin pathways, which are significantly disrupted during sleep deprivation but can be partially restored through brief sleep episodes. The modest recovery in physical performance suggests that 45 min naps are insufficient for the complete restoration of the physiological systems underlying anaerobic power output. This implies that while neural fatigue can be alleviated relatively quickly with short sleep periods, muscular and metabolic recovery requires longer rest periods or alternative interventions to achieve optimal performance. This is likely due to the need for protein synthesis, glycogen replenishment, and clearance of inflammatory markers, which cannot be completed within the limited timeframe of a 45 min nap [5]. The counterintuitive decrease in depression scores during TSD likely reflects emotional blunting rather than genuine mood improvement, which is consistent with previous research showing reduced emotional reactivity during severe sleep deprivation [89]. This interpretation is supported by the concurrent increase in other negative mood indicators that exhibit the expected effects of sleep deprivation.

### 4.4. Comparison with Previous Research

Our findings closely align with those of the existing literature and offer new insights into combat sports. The observed improvements in cognitive performance, marked by a 14.1% increase in accuracy and a 16.1% enhancement in reaction time, are consistent with [50], who demonstrated that 30 min naps enhance cognitive outcomes and effectively mitigate cognitive decline in athletic populations. However, our findings revealed larger effect sizes than those previously reported, which may be attributed to several factors that are unique to our study population and methodology. First, the elite training status of Kung-Fu athletes may have enhanced their sleep efficiency and recovery capacity, leading to more pronounced benefits from strategic napping [19,90]. Second, the specific cognitive demands of combat sports, which require rapid decision-making under pressure, may be particularly sensitive to sleep deprivation, thereby exhibiting greater improvement after recovery interventions [18,91]. Additionally, this discrepancy may result from sleep inertia effects, as naps exceeding 30 min often induce slow-wave sleep, potentially causing transient performance decrements lasting 20–30 min post-awakening [40]. The findings on interaction effects contribute novel insights to the literature by demonstrating that the benefits of napping are inconsistent across different performance conditions. Our data reveal effect sizes that are 3–10 times larger under conditions of sleep deprivation than when individuals are well-rested, providing quantitative evidence for the compensatory nature of napping interventions. This challenges the common assumption that napping universally enhances performance, supporting a more nuanced understanding that napping primarily replenishes depleted physiological and cognitive resources rather than enhancing normal performance capacity [92].

### 4.5. Theoretical Implications

The present findings make a substantial contribution to the theoretical framework of sleep science, prompting the necessary revisions to existing models. First, they support the differential recovery hypothesis by demonstrating that different performance domains recover at varying rates after sleep deprivation. This study advances this hypothesis by introducing a hierarchical model of recovery. In this model, cognitive function recovers most rapidly within 45 min, followed by mood states with an intermediate recovery time, and physical performance, which takes the longest to restore. This hierarchy may imply an evolutionary prioritisation of cognitive functions, which are crucial for survival and decision-making, over purely physical capabilities [5].

Second, our results enhance the two-process model of sleep regulation [93] by showing that brief sleep intervals effectively reduce homeostatic sleep pressure for cognitive functions but are insufficient for complete physiological recovery in healthy adults. Our findings further support this hypothesis by presenting a hierarchical recovery model. In this model, cognitive function recovered most swiftly within 45 min, followed by mood states with an intermediate recovery time, whereas physical performance required the longest duration for its restoration. These findings suggest a modification of the traditional two-process model to include domain-specific homeostatic drives, in which cognitive and physical performance may be regulated by partially independent sleep mechanisms [93].

Third, our significant interaction effects provide strong empirical support for compensatory sleep theory while extending its relevance to athletic populations. We propose that the compensatory benefits of napping follow a dose–response relationship influenced by the extent of sleep debt, specific performance domain assessed, and individual characteristics of athletes. This expanded theoretical framework suggests that optimal napping protocols should be tailored to these factors rather than relying on universal recommendations [4].

### 4.6. Practical Implications

The findings of this study have profound implications for improving athletic performance and ensuring the safety of athletes. For athletes engaged in combat sports, we suggest adopting the following evidence-based napping protocol: (1) schedule 45 min naps between 13:00 and 15:00 to align with natural circadian dips in alertness, while avoiding interference with nighttime sleep; (2) establish standardised napping environments with controlled temperatures (18–21 °C), minimal light exposure (<10 lx), and the use of white noise or earplugs to enhance sleep quality; (3) implement a structured awakening protocol that includes 5 min of light exposure (>1000 lx) followed by 15 min of light physical activity to mitigate the effects of sleep inertia; and (4) ensure at least a 60 min gap between the end of a nap and critical performance tasks to allow for the full dissipation of sleep inertia [40].

For coaches and sports scientists, these findings advocate the creation of periodized napping strategies that align with training and competition schedules. During high-intensity training blocks or competition periods characterised by sleep restriction, strategic napping should be prioritised as the primary recovery intervention. Conversely, during well-rested periods, it may be more beneficial to allocate training time to other performance-enhancement strategies, given the minimal benefits observed from routine napping under non-sleep-deprived conditions. These findings endorse strategic napping protocols for shift workers, emergency responders, military personnel, and healthcare professionals who frequently experience sleep deprivation. Implementation should include (1) organisational policies that provide dedicated napping facilities and protected time for strategic napping, (2) education programmes that teach optimal napping techniques and timing, and (3) individual assessment tools to identify workers who would benefit the most from napping interventions based on their sleep debt and performance requirements.

### 4.7. Study Limitations

Several methodological limitations significantly impact the internal and external validity of the findings. First, the sample exclusively comprised young, healthy male athletes (*n* = 24), which fundamentally limits the generalisability of our findings to female athletes, older populations, and individuals with sleep disorders, as well as to non-elite populations and other sports disciplines with different competitive demands and recovery profiles. This limitation is particularly significant given the emerging evidence of sex differences in sleep architecture, nap responsiveness, and recovery patterns. Female athletes may demonstrate different napping benefits owing to hormonal fluctuations that affect sleep quality and recovery capacity during their menstrual cycles [94]. The age homogeneity of our sample also limits its applicability to master athletes or recreational participants, who may have different sleep needs and recovery capacities.

Second, although the laboratory setting ensured experimental control, it created an artificial environment that may not reflect real-world conditions. Athletes typically nap in varied environments with different noise levels, comfort, and privacy, which can significantly affect their sleep quality and subsequent performance benefits. Controlled laboratory conditions may have optimised napping effectiveness in ways that cannot be replicated in practical settings, potentially overestimating the benefits of strategic napping in applied contexts [92].

Third, the fixed 45 min nap duration, based on previous research, may not represent the optimal recovery period for all participants. Individual differences in sleep architecture, particularly variations in sleep onset latency and slow-wave sleep propensity, could result in some participants achieving minimal actual sleep, whereas others experienced deep sleep stages. This limitation may have introduced significant interindividual variability that was not adequately captured in our analysis, potentially masking important individual response patterns that could inform personalised napping recommendations [95].

Fourth, our reliance on visual observation for sleep monitoring during nap periods represents a significant methodological limitation that affects the internal validity of our dose–response interpretations because the absence of actigraphy or polysomnography prevents the verification of actual sleep duration and sleep stage composition during the nap, which may influence the interpretation of nap-related effects.

### 4.8. Future Research Directions

Based on the most significant findings, uncertainties, and limitations of our study, we propose the following prioritised research agenda. First, immediate priority should be given to dose–response studies that explore various nap durations (10, 20, 30, 45, and 60 min) with polysomnographic monitoring or actigraphy to establish optimal napping protocol. These studies should include standardised post-awakening intervals (30, 60, and 90 min) to precisely quantify the effects of sleep inertia and determine the minimum recovery time required before resuming training or competition activities. Second, high-priority longitudinal studies examining repeated napping protocols over extended training periods (8–12 weeks) should investigate the cumulative benefits, potential adaptation effects, and individual factors predicting nap responsiveness. These studies should incorporate chronotype assessments and baseline sleep quality measures to develop predictive models for identifying athletes who are most likely to benefit from strategic napping. Third, field studies in competitive settings are critical for addressing the ecological validity of laboratory-based findings. These studies should evaluate practical implementation challenges, including facility requirements, timing constraints, and integration with existing recovery protocols during competitions. Special attention should be paid to cultural factors and sport-specific demands that may influence the acceptability and effectiveness of napping [50]. Fourth, mechanistic research using advanced neuroimaging techniques (fMRI and EEG), cortisol and inflammatory marker assessment, and metabolomic analysis should clarify the neurophysiological pathways underlying the differential recovery patterns across performance domains. This research is essential for developing targeted interventions that maximise cognitive recovery while addressing slower physical recovery processes. Fifth, studies involving female athletes and diverse athletic populations should assess the generalisability of and potential sex differences in napping effectiveness. These studies should specifically examine how hormonal fluctuations, menstrual cycle phase, and contraceptive use influence the benefits of napping, as these factors may significantly modulate recovery responses. Additionally, future studies should incorporate aerobic performance outcomes using gold-standard assessments (e.g., cardiopulmonary exercise testing, CPET) to determine whether nap-related benefits extend to aerobic capacity and broader health-related outcomes [96].

## 5. Conclusions

This study provides compelling evidence that strategic napping effectively counters the performance decline caused by sleep deprivation in elite combat sports athletes, offering differential benefits across the cognitive, mood, and physical performance domains. Substantial cognitive recovery (14–16% improvement) and mood restoration (53.8% vigour recovery) demonstrated the practical value of 45 min naps for maintaining performance capacity under sleep restriction conditions. The significant interaction effects between sleep and napping conditions indicate that the benefits of napping are most pronounced under elevated sleep pressure, supporting strategic rather than routine implementation and fundamentally challenging universal napping recommendations.

While physical performance showed modest recovery, rapid cognitive restoration suggests that napping strategies should prioritise decision-making and attentional capacity in competitive contexts requiring cognitive–motor integration, particularly in combat sports, where split-second tactical decisions determine performance outcomes. These findings advance both the theoretical understanding of differential recovery mechanisms and the practical application of evidence-based recovery strategies. For the broader field of sleep science and sports performance, this study underscores the importance of domain-specific recovery strategies and provides empirical support for strategic napping as a targeted intervention for optimising cognitive performance in high-demand athletic environments, while highlighting the need for complementary interventions to address physical performance recovery.

## Figures and Tables

**Table 1:** Participant demographics and training characteristics (*n* = 24).

Characteristic	Value
Age (years)	20.67 ± 1.76
Height (m)	1.71 ± 0.04
BMI (kg/m^2^)	21.4 ± 2.1
Training experience (years)	12.8 ± 2.1
Sex	Male
Chronotype	Neutral

**Table 2:** Profile of Mood States (POMS).

*Outcome Measure*	TSD − N	TSD + NAP	WSD − NAP	WSD + NAP	*F* (Sleep)	*p* (Sleep)	*ηp*^2^ (Sleep)	*F* (Nap)	*p* (Nap)	*ηp*^2^ (Nap)	*F* (Sleep × Nap)	*p* (Sleep × Nap)	*ηp*^2^ (Sleep × Nap)
**Anger**	15.8 ± 2.90	8.44 ± 1.96	12.36 ± 7.58	10.24 ± 7.38	0.48	0.50	0.02	23.14	0.001	0.49	9.37	0.005	0.28
**Depression**	4.00 ± 3.75	2. 84 ± 1.18	9.76 ± 8.07	10.28 ± 8.61	29.99	0.001	0.55	0.10	0.75	0.004	0.57	0.46	0.023
**Tension**	15.60 ± 2.10	9.32 ± 2.08	9.36 ± 4.99	9.32 ± 4.53	15.32	0.001	0.39	19.15	0.001	0.44	26.30	0.001	0.52
**Confusion**	14.32 ± 1.89	9.16 ± 1.70	7.00 ± 3.52	7.32 ± 3.39	88.11	0.001	0.79	17.24	0.001	0.42	25.51	0.001	0.52
**Vigour**	9.48 ± 2.24	10.72 ± 0.46	21.20 ± 2.69	22.48 ± 3.63	66.58	0.001	0.97	5.95	0.02	0.20	0.001	0.97	0.000
**Fatigue**	17.20 ± 2.74	6.68 ± 1.57	5.24 ± 4.00	4.52 ± 4.10	90.31	0.001	0.79	114.41	0.001	0.83	64.03	0.001	0.73

**Note.** TSD − N = Total sleep deprivation without napping; TSD + NAP = Total sleep deprivation with napping; WSD − NAP = Without sleep deprivation and without napping; WSD + NAP = Without sleep deprivation with napping.

**Table 3:** Subjective Perceptions of Sleepiness and Recovery.

*Outcome Measure*	TSD − N	TSD + NAP	WSD − NAP	WSD + NAP	*F* (Sleep)	*p* (Sleep)	*ηp*^2^ (Sleep)	*F* (Nap)	*p* (Nap)	*ηp*^2^ (Nap)	*F* (Sleep × Nap)	*p* (Sleep × Nap)	*ηp*^2^ (Sleep × Nap)
**Sleep Quality**	1.00 ± 0.00	1.08 ± 0.28	4.12 ± 0.78	3.92 ± 0.73	474.04	0.001	0.95	0.38	0.543	0.016	2.76	0.11	0.10
**Fatigue**	87.80 ± 10.81	62.60 ± 13.78	12.00 ± 7.91	12.00 ± 8.54	975.59	0.001	0.98	30.06	0.001	0.56	29.14	0.001	0.55
**Sleep Quantity**	8.30 ± 1.73	0.00 ± 0.00	8.00 ± 1.46	7.78 ± 1.75	411.89	0.001	0.94	229.64	0.001	0.95	88.29	0.001	0.89
**Concentration**	16.80 ± 9.45	34.80 ± 11.13	87.00 ± 11.55	87.80 ± 10.21	641.61	0.001	0.96	35.26	0.001	0.56	14.37	0.001	0.37
**Attention**	15.40 ± 7.63	34.20 ± 14.34	88.00 ± 12.16	86.60 ± 10.77	951.78	0.001	0.98	20.87	0.001	0.47	18.04	0.001	0.43

**Note.** TSD − N = Total sleep deprivation without napping; TSD + NAP = Total sleep deprivation with napping; WSD − NAP = Without sleep deprivation and without napping; WSD + NAP = Without sleep deprivation with napping.

**Table 4:** Decision-making.

*O* *utcome Measure*	TSD − N	TSD + NAP	WSD − NAP	WSD + NAP	*F* (Sleep)	*p* (Sleep)	*ηp*^2^ (Sleep)	*F* (Nap)	*p* (Nap)	*ηp*^2^ (Nap)	*F* (Sleep × Nap)	*p* (Sleep × Nap)	*ηp*^2^ (Sleep × Nap)
**DT (s)**	0.92 ± 0.13	0.78 ± 0.09	0.58 ± 0.08	0.60 ± 0.08	88.06	0.001	0.786	42.99	0.001	0.642	47.42	0.001	0.64
**DA (%)**	18.44 ± 2.24	21.04 ± 2.23	24.00 ± 2.16	23.40 ± 2.06	131.67	0.001	0.85	26.67	0.001	0.53	50.36	0.001	0.68

**Note.** DT: Decision Time, DA = Decision Accuracy. TSD − N = Total sleep deprivation without napping; TSD + NAP = Total sleep deprivation with napping; WSD − NAP = Without sleep deprivation and without napping; WSD + NAP = Without sleep deprivation with napping.

**Table 5:** Barrage test.

*O* *utcome Measure*	TSD − N	TSD + NAP	WSD − NAP	WSD + NAP	*F* (Sleep)	*p* (Sleep)	*ηp*^2^ (Sleep)	*F* (Nap)	*p* (Nap)	*ηp*^2^ (Nap)	*F* (Sleep × Nap)	*p* (Sleep × Nap)	*ηp*^2^ (Sleep × Nap)
**Barrage Score**	49.60 ± 11.32	56.68 ± 13.70	65.36 ± 9.82	62.28 ± 12.18	69.70	0.001	0.74	0.82	0.37	0.033	11.97	0.002	0.33

**Note.** Higher scores indicate better performance. TSD − N = Total sleep deprivation without napping; TSD + NAP = Total sleep deprivation with napping; WSD − NAP = Without sleep deprivation and without napping; WSD + NAP = Without sleep deprivation with napping.

**Table 6:** Physical Performance.

*Outcome Measures*	TSD − N	TSD + NAP	WSD − NAP	WSD + NAP	*F* (Sleep)	*p* (Sleep)	*ηp*^2^ (Sleep)	*F* (Nap)	*p* (Nap)	*ηp*^2^ (Nap)	*F* (Sleep × Nap)	*p* (Sleep × Nap)	*ηp*^2^ (Sleep × Nap)
**HJ (m)**	2.12 ± 0.21	1.92 ± 0.18	2.08 ± 0.21	2.10 ± 0.21	12.63	0.002	0.35	76.25	0.001	0.76	56.80	0.001	0.70
**VJ (cm)**	33.23 ± 6.03	34.02 ± 6.14	37.19 ± 6.47	36.45 ± 5.94	35.68	0.001	0.60	0.006	0.94	0.000	5.68	0.03	0.19
**ILMS (kg)**	110.10 ± 19.79	113.36 ± 23.47	117.90 ± 27.87	124.30 ± 27.88	10.59	0.003	0.31	2.73	0.11	0.10	0.17	0.68	0.01
**IBMS (kg)**	121.48 ± 30.01	135.32 ± 36.82	142.58 ± 43.94	137.42 ± 38.08	5.95	0.022	0.20	1.68	0.21	0.07	5.95	0.02	0.20
**SMBT (m)**	4.40 ± 0.69	4.04 ± 0.63	4.47 ± 0.70	4.39 ± 0.66	16.52	0.001	0.41	103.06	0.001	0.81	31.50	0.001	0.57

**Note.** HJ, Horizontal Jump, VJ = Vertical Jump, ILMS = Isometric Leg Muscle Strength, IBMS = Isometric Back Muscle Strength, SMBT = Seated Medicine Ball Throw. TSD − N = Total sleep deprivation without napping; TSD + NAP = Total sleep deprivation with napping; WSD − NAP = Without sleep deprivation and without napping; WSD + NAP = Without sleep deprivation with napping.

## Data Availability

All datasets used and/or analysed during the current study are available from the corresponding author upon reasonable request.
